# Addition of new neurons and the emergence of a local neural circuit for precise timing

**DOI:** 10.1371/journal.pcbi.1008824

**Published:** 2021-03-17

**Authors:** Yevhen Tupikov, Dezhe Z. Jin

**Affiliations:** Departments of Physics and Huck Institutes of the Life Sciences, Pennsylvania State University, University Park, Pennsylvania, United States of America; University of Pittsburgh, UNITED STATES

## Abstract

During development, neurons arrive at local brain areas in an extended period of time, but how they form local neural circuits is unknown. Here we computationally model the emergence of a network for precise timing in the premotor nucleus HVC in songbird. We show that new projection neurons, added to HVC post hatch at early stages of song development, are recruited to the end of a growing feedforward network. High spontaneous activity of the new neurons makes them the prime targets for recruitment in a self-organized process via synaptic plasticity. Once recruited, the new neurons fire readily at precise times, and they become mature. Neurons that are not recruited become silent and replaced by new immature neurons. Our model incorporates realistic HVC features such as interneurons, spatial distributions of neurons, and distributed axonal delays. The model predicts that the birth order of the projection neurons correlates with their burst timing during the song.

## Author summary

Functions of local neural circuits depend on their specific network structures, but how the networks are wired is unknown. We show that such structures can emerge during development through a self-organized process, during which the network is wired by neuron-by-neuron recruitment. This growth is facilitated by steady supply of immature neurons, which are highly excitable and plastic. We suggest that neuron maturation dynamics is an integral part of constructing local neural circuits.

## Introduction

During development, the birth order of neurons plays a critical role in constructing the brain’s large-scale structures. In mammalian cortex, neurons that are destined to the deep cortical layers are born earlier than those to the superficial layers [1, 2]. In rodent hippocampus, early born neurons and late born neurons form distinctive parallel circuits through the hippocampal pathway [3]. However, whether birth order is also important in constructing microcircuits in local brain areas is unknown [4]. The premotor nucleus HVC (proper name) of the zebra finch provides an excellent opportunity to investigate this issue.

**Fig 1:**
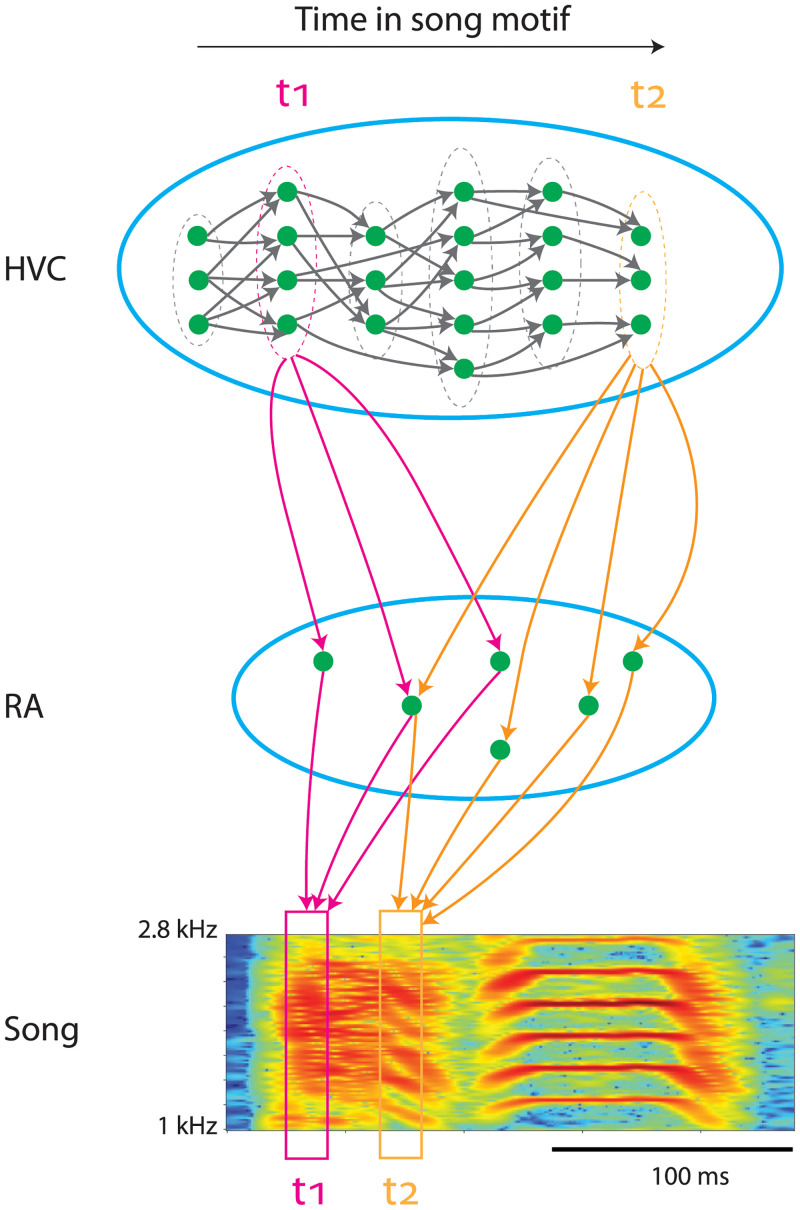
Song production circuit. HVC controls timing of acoustic features in a song motif. Time is encoded with a feedforward synaptic chain network, which supports propagation of spiking activity (green dots, HVC_RA_ neurons; grey arrows, local synaptic connections; dotted ovals, simultaneously firing groups of neurons). Each HVC_RA_ neuron bursts once at a precise time. Groups of simultaneously firing projection neurons encode moments of time (two time points *t*_1_ and *t*_2_ are illustrated). Through the projection patterns, the groups drive specific sets of RA neurons, which in turn drive specific acoustic features of the song at the specific moments (spectrogram of a zebra finch song syllable is shown). Two sets of projection patterns, magenta arrows at *t*_1_ and orange arrows at *t*_2_, are shown as examples. The HVC to RA projection patterns are learned through reinforcement learning. The synaptic chain network in HVC serves as the backbone that provides the timing structure for the reinforcement learning.

HVC is a premotor nucleus that drives singing of the courtship song in the zebra finch [5, 6] (Fig 1). An adult zebra finch sings repetitions of a song motif consisting of a fixed sequence of syllables [7]. The excitatory HVC neurons that project to the downstream premotor area RA (robust nucleus of the arcopallium) encode the timing of acoustic features of the song [8]. Each RA-projecting HVC (HVC_RA_) neuron bursts once during the motif [8, 9]. As a population, HVC_RA_ neurons sequentially burst throughout the entire motif, including the silent gaps between the syllables [10, 11].

HVC_RA_ neurons that simultaneously burst at a single time point drive a specific set of RA neurons at that moment, which in turn activates downstream motor neurons and produces a vocalization of the specific acoustic feature in the song motif (Fig 1). The projection patterns from HVC_RA_ neurons to RA neurons thus encode a specific learned song. These patterns are set in the critical period of a male juvenile zebra finch (∼90 days post hatch (dph)), who practices to match his own song to the song template that he memorized after hearing the tutor’s song [7]. This song learning process is achieved through reinforcement learning, during which the connections from HVC_RA_ neurons to RA neurons are established through trial-and-error [12–15]. Song development progresses in four stages [16, 17]: subsong, which is highly variable and structureless (∼48 dph); protosyllable song, which contains syllables with definable durations but undistinguishable acoustic features (∼58 dph); multi-syllable song, which contains syllables with definite durations and distinctive spectral characteristics (∼62 dph); and motif song, which consists of a reliable sequence of stereotypical syllables like the adult song (∼73 dph).

There is strong evidence that the sequential bursting of HVC_RA_ neurons is generated within HVC [9, 18–21]. Moreover, HVC_RA_ neurons most likely form a feedforward synaptic chain network (Fig 1) [9, 21, 22]. The feedforward network supports propagation of bursting activity of HVC_RA_ neurons, and each HVC_RA_ neuron bursts once at a precise time, as observed experimentally [8, 9]. Such a microcircuit in HVC acts as the infrastructure for the reinforcement learning of a specific song. Therefore, the synaptic chain network in HVC must be wired up before the reinforcement learning can proceed.

HVC_RA_ neurons are born and added to HVC mostly after hatching [23–26]. In the zebra finch, the number of HVC_RA_ neurons almost doubles from 20 to 50 days post hatch [27], a period that overlaps with the subsong and the protosyllable song stages [16, 17]. This is unlike two other major neuron types in HVC: most GABA (*γ*-Aminobutyric acid)-ergic interneurons (HVC_INT_ neurons) and neurons that project to area X (HVC_X_ neurons) are already in HVC before hatching [24] (but see [25]). Therefore, HVC_RA_ neurons have a wide range of birthdates before the emergence of the multi-syllable song and the song motif.

Previous computational models [28, 29] and single unit recordings in juvenile zebra finches [17] have suggested that the feedforward synaptic chain network in HVC forms through growth by gradual recruitment of HVC_RA_ neurons to the network. However, these earlier works did not address whether the ongoing neurogenesis in juvenile zebra finches plays any role. Indeed, although neurogenesis in HVC post hatch has been observed for decades, its role for song learning in the zebra finch has remained a mystery [26, 30].

In this paper, we propose that the constant supply of newborn HVC_RA_ neurons plays a crucial role in building the synaptic chain network in HVC. We investigate this hypothesis through a computational model that builds on the previous models of network growth in HVC [28, 29]. Like these earlier computational models, we propose that the synaptic chain network is wired through repeated activations of a set of HVC_RA_ neurons that act as the training neurons; spontaneous activity of the neurons; and a set of synaptic plasticity rules that shape the connectivity between HVC_RA_ neurons. The synaptic chain network grows by gradual recruitment of neurons into the network. However, our model incorporates more biologically realistic features, including explicit incorporation of HVC_INT_ neurons rather than simplifying the inhibitory actions as idealized global inhibition between HVC_RA_ neurons; implementation of axonal delays between HVC_RA_ neurons, which has shown to be substantial and is important for determining the connectivity structure of the synaptic chain network [21]; and spatial structure of HVC_RA_ connectivity, which has been recently measured in the zebra finch [20]. Most importantly, the maturation dynamics of HVC_RA_ neurons is modeled.

Newly born neurons have a number of properties that distinguish them from mature neurons. Immature neurons in rodents [31–33] and in songbird HVC [34] are more excitable; and in rodents, they are more amenable to synaptic plasticity [35]. In adult rodent hippocampus, these properties make adult-born dentate gyrus neurons more likely to participate in new memory formation than mature neurons [36]. We propose that newly born neurons in HVC similarly facilitate the growth of synaptic chain network. In our model, the synaptic chain network grows through the spontaneous activity of neurons. Due to their high excitability, we propose that newly added HVC_RA_ neurons are preferentially recruited at the growth edge of the network. We suggest that these neurons mature fast after incorporation into the network due to consistent activations, and they form a new edge of growth and recruit a new cohort of immature neurons. This process iterates, creating a synaptic chain network that supports precisely timed sequential bursting of HVC_RA_ neurons when the training neurons are activated. We predict that the timing of the bursts relative to the onset of the activity by the training neurons correlates with the birth order of HVC_RA_ neurons during the wiring process.

We show evidence that the maturity of HVC_RA_ neurons correlates with their timing in song syllables by reanalyzing the data from the previous experiments on juvenile zebra finch [17]. We also show that our model creates the observed spatial distribution profile for the connections between HVC_RA_ neurons [20]. With a wide delay distribution between these connections, as observed by experiments [21], our model produces a robust polychronous chain network with continuous and precise time representation, which is recently proposed to be the structure of the synaptic chain network in HVC [21]. The previous models of chain growth neglected synaptic delays and produced synfire chains [28, 29], in which a postsynaptic neuron receives synchronous inputs, and the presynaptic neurons that provide these inputs fire synchronously as well [37]. In a polychronous chain network, the inputs also arrive synchronously at the postsynaptic neuron. However, the presynaptic neurons fire asynchronously due to the distributed axonal delays [21, 38]. Our model additionally predicts that HVC_RA_ neurons in the growing chain network receive less forward inhibition from the HVC_RA_ neurons that drive them, which was not predicted by the previous models due to the omission of HVC_INT_ neurons [28, 29].

## Results

### Maturation dynamics of HVC_RA_ neurons

**Fig 2:**
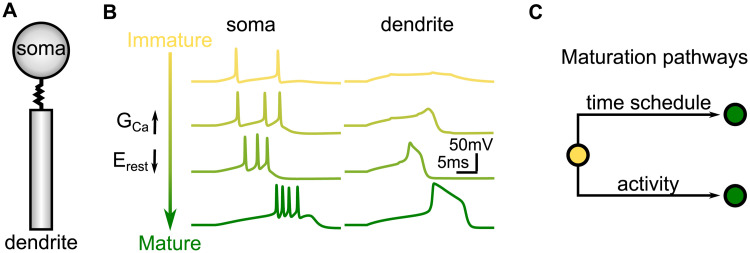
Computational model of HVC_RA_ neurons and the maturation process. A: An HVC_RA_ neuron is modeled as two-compartmental Hodgkin-Huxley with soma and dendrite. B: Responses of HVC_RA_ neurons to current injection to the dendritical compartment at different maturation stages. C: Two pathways for neuronal maturation: scheduled maturation under spontaneous activity, and accelerated maturation driven by activity when neuron spikes reliably.

To investigate the possible role of newly born immature HVC_RA_ neurons in wiring the HVC network, we created a computational model of the maturation dynamics of these neurons. We modeled HVC_RA_ neurons using the two-compartmental Hodgkin-Huxley neurons with soma and dendrite (Fig 2A), following the previous models [9, 22, 39]. The somatic compartment contains sodium, delayed-rectifying potassium, and low-threshold potassium currents for generating sodium spikes. The dendritic compartment contains calcium and calcium-activated potassium currents that, in mature neurons, can generate dendritic spikes that drive stereotypical tight bursts of sodium spikes in the somatic compartment.

This model was modified for new immature HVC_RA_ neurons. The resting membrane potential is set higher by 25 mV, since it was generally observed in rodents [32] and in HVC [34] that the resting membrane potentials of immature neurons are higher than that of mature neurons. The calcium conductance is set to zero to reflect “weak” dendritic compartment in new immature neurons. Hence, a new immature neuron is incapable of generating tight bursts (Fig 2B).

With age and activity, a new immature neuron gradually matures. During the maturation, the resting potential is gradually decreased and the calcium conductance is gradually increased in the dendritic compartment, eventually reaching the values for the mature neurons. Dendritic calcium spike and tight burst of somatic sodium spikes gradually emerges during this process (Fig 2B). The time course of maturation is age and activity dependent in our model (Fig 2C). Due to the elevated resting potential and noise, new immature neurons spike spontaneously at ∼ 0.6 Hz. A spontaneously active immature neuron matures following a time schedule, according to which both the resting membrane potential and the calcium conductance exponentially approach their mature values with a time constant of 50,000 s. When a neuron is recruited into the network and spikes reliably, the maturation progresses with a faster rate, with the time constant set to 500 s. In our model, the spontaneous activity decreases with age, practically disappearing in mature neurons (S1 Fig). Therefore, neurons that do not get recruited to the network gradually become silent. The silent neurons were replaced by new immature neurons in our model to mimic the continuous addition and death of HVC_RA_ neurons in juvenile zebra finch [40].

### Initial HVC network

Among the three major HVC neuron types, HVC_X_ neurons were shown to impact minimally on song production in a laser ablation study [41]. Furthermore, analysis of HVC connectivity suggested that HVC_RA_ neurons excite HVC_X_ neurons, but HVC_X_ neurons rarely connect back to HVC_RA_ neurons [42]. These results suggest that HVC_X_ neurons are not necessary for song production. Therefore, we did not include HVC_X_ neurons in our model.

**Fig 3:**
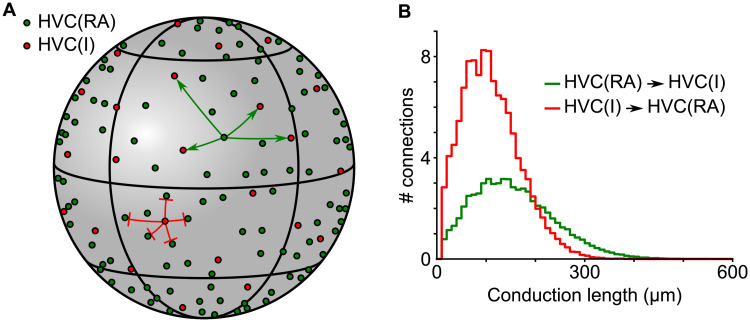
Schematic of a network arrangement and connectivity. A: HVC_RA_ (dark green circles) and HVC_INT_ (red circles) neurons are distributed over the surface of a sphere. HVC_INT_ neurons form a lattice-like pattern, while HVC_RA_ neurons are distributed randomly. Examples of connections from one HVC_RA_ neuron to HVC_INT_ neurons and from one HVC_INT_ to HVC_RA_ neurons are shown. B: Distribution of axonal conduction lengths for connections between HVC_RA_ and HVC_INT_ neurons.

HVC of the zebra finch is roughly an ellipsoidal structure with axial dimensions 2000 *μ*m, 500 *μ*m and 500 *μ*m [20]. There are approximately 20,000 song-related HVC_RA_ neurons and 5,500 HVC_INT_ neurons [12, 43]. Due to the limitation of computational power, we could not include this many neurons in our model. Instead, we restricted ourselves to 2000 HVC_RA_ and 550 HVC_INT_ neurons. The connections between HVC_RA_ neurons and HVC_INT_ neurons were set using a simple distance based probabilistic rule suggested by the experiments [20]. Since the number of neurons is small in our simulations, distributing them in the HVC-sized ellipsoidal space creates inhomogeneity in the connectivity between HVC_RA_ neurons and HVC_INT_ neurons, such that the HVC_RA_ neurons near the center are connected with more HVC_INT_ neurons than those off the center and even more so than those near the edge. It is possible to resolve this issue by modifying the connectivity rule such that the locations of HVC_RA_ neurons is also a factor. However, we chose to simplify the problem and placed neurons on a 2D sphere of radius 260 *μ*m. This eliminated the boundary effects on the connectivity between HVC_RA_ neurons and HVC_INT_ neurons. HVC_INT_ neurons were placed in a lattice-like grid on the sphere, and HVC_RA_ neurons randomly (Fig 3A). The distance between neighboring HVC_INT_ neuron roughly matches the value estimated from the volume and the number of HVC_INT_ neurons in the zebra finch HVC. We created connections between HVC_RA_ and HVC_INT_ neurons probabilistically according to the Gaussian distributions based on the distance between the neurons (Fig 3B). These distributions are similar to those observed in experiments [20]. On average, an HVC_RA_ neuron connects to 65 HVC_INT_ neurons with mean distance 155 *μ*m, and an HVC_INT_ neuron connects to 115 HVC_RA_ neurons with mean distance 110 *μ*m. Initially, all HVC_RA_ neurons were new immature neurons and there were no connections between them.

We also created axonal time delays between all neurons by setting the conduction velocity to 100 *μ*m/ms (the value observed in HVC [21]) and using the distances between the neurons on the sphere. The range of the computed axonal delays between HVC_RA_ neurons in the model approximately matched the measured values in zebra finch HVC (1 to 7.5 ms) [21].

### Growth of synaptic chain network

**Fig 4:**
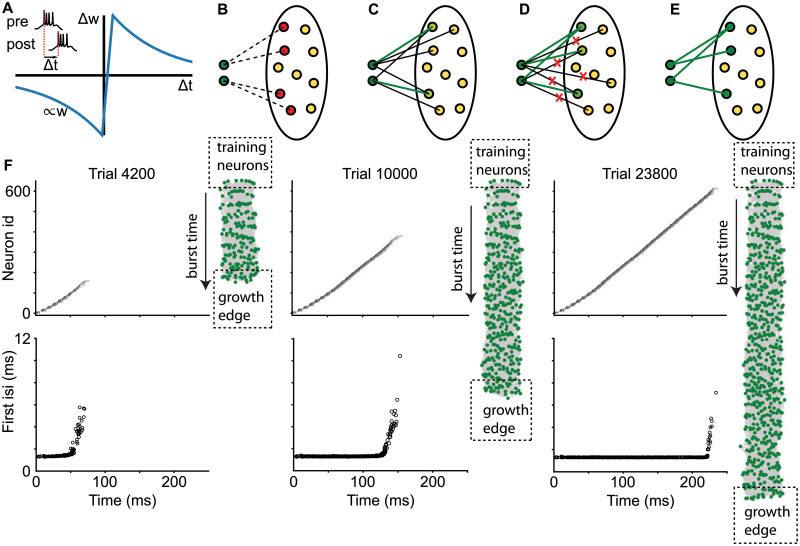
Mechanism of network growth. A: The burst-timing dependent plasticity (BTDP) rule is based on the timing between burst onsets of HVC_RA_ neurons. B-E: Schematic of the recruitment mechanism. B: Network growth begins with the training neurons (dark green circles) activated in each simulation trial and other HVC_RA_ neurons being immature (yellow circles). Silent connections (dashed lines) emerge from the training neurons to the spontaneously active immature HVC_RA_ (red circles) according to the BTDP rule. C: Some silent connections randomly become active (black lines), undergo further strengthening, and become strong supersynaptic connections (thick green lines). D: When the training neurons acquire certain number of strong supersynaptic connections, other weak connections are pruned (red crosses). E: The recruited neurons (dark green circles) spike reliably after the training neurons and begin to recruit immature neurons to the network. F: Network growth is a gradual process in which immature HVC_RA_ neurons are added to the end of the growth edge. Spike raster plots (top row) and first interspike intervals (bottom row) at different training trials are shown. Also shown are the network topology, in which green dots are neurons in the synaptic chain network and gray lines are the connections between the neurons. The green dots on top are the training neurons, and those at the bottom are the newly recruited neurons. The burst times of the neurons approximately align with their positions in the network from the top to the bottom. The first interspike intervals of the neurons bursting at the end of the spike sequence are larger than those bursting earlier (bottom role), showing that immature neurons are attached to the growth edge.

To grow a network of connected HVC_RA_ neurons, we used a combination of a Hebbian-like burst-timing dependent plasticity (BTDP) (Fig 4A) and two additional plasticity rules for HVC_RA_ neurons—axon remodeling and potentiation decay, which are similar to those used in the previous models for growth of synaptic chain networks [28, 29].

The BTDP is modified from the spike-timing dependent plasticity (STDP) rule widely observed in variety of brain areas in many species [44]. Specifically, the time difference Δ*t* between the first spikes of the post- and the pre-synaptic neurons is used. When Δ*t* > 2 ms, the synapse is potentiated (long-term potentiation, or LTP); when Δ*t* < 2 ms, the synapse is depressed (long-term depression, or LTD). Here we introduced a small positive shift to the LTD window. This ensures that no connections emerge between neurons that fire synchronously. The magnitude of LTP induction is maximum at Δ*t* = 5 ms, and LTD is maximal at Δ*t* = −1 ms (Fig 4A). The magnitudes of both LTP and LTD induction decay exponentially as the absolute value of Δ*t* increases (decay constant 30 ms).

We distinguished three types of connections between HVC_RA_ neurons, depending on their strengths. Silent synapses are weak, nonfunctional connections, with synaptic conductance smaller than a threshold value *W*_*a*_. They correspond to the synapses containing only NMDA receptors and do not elicit response in the postsynaptic neuron [45]. When synaptic strengths exceed *W*_*a*_, the synapses become active and produce depolarizations in the postsynaptic neurons. Strong connections with weights above *W*_*s*_ are considered as supersynaptic (or strong) connections. The supersynapses correspond to the synapses that have large and stable spine heads and are maintained through active protein synthesis after repeated LTP inductions [46].

We randomly selected a set of 10 HVC_RA_ neurons as the training neurons, which formed a seed for the network growth. The training neurons were made fully mature with adult values for the resting potential and calcium dendritic conductance. HVC_RA_ neurons that were not in the training set, called pool neurons, started as new immature neurons with high resting potential and devoid of dendritic calcium channels.

One simulation trial lasted for 500 ms in network dynamics. At each trial, the training neurons were stimulated with a synchronous kick of strong excitatory conductance. To ensure that the growth dynamics was not influenced by the initial state of the network, the stimulation was delivered at a random time between 100 to 400 ms in each trial. Immature pool neurons were spontaneously active during the trials due to the elevated resting potentials and noisy fluctuations in the membrane potentials. When some pool neurons spiked after the training neurons, silent connections from the training neurons to the pool neurons emerged according to the BTDP rule (Fig 4B). During the repeated trials, silent synapses stochastically changed their strength via LTP and LTD, and could randomly become active (Fig 4C). Emergence of too many active connections leads to uncontrolled network growth and runaway network activity [28, 29]. To avoid this, we introduced potentiation decay for all synapses, following what was done in the previous models [28, 29]. Specifically, synaptic weights of all synapses were decreased by a constant value *δ* at the end of each trial.

Depolarizations of the pool neurons provided by the active synapses from the training set biased these neurons to be more active during the subsequent trials. Thus, a positive feedback emerged, since the activity of these pool neurons facilitated strengthening of the synapses via LTP, eventually forming supersynaptic connections from the training neurons. To enforce sparse output connections, we only allowed each HVC_RA_ neuron to make a limited number of supersynaptic connections to other HVC_RA_ neurons, which was set to 10 in the model. When a neuron acquired the maximal number of supersynaptic outputs, the neuron underwent axon remodeling, in which other weak outgoing connections were pruned and did not affect their postsynaptic targets anymore [28, 29] (Fig 4D and 4E). Limitations on the number of the strong outputs created a competition between the pool neurons for the convergent inputs from the training set. When the training neurons formed the allowed number of supersynaptic connections, their postsynaptic targets were spiking reliably in each trial. After this point, the training neurons did not recruit any more targets because of the limitation of the supersynapses they could form. This restriction is also necessary for avoiding the “hoarding problem”, in which the training neurons make strong connections to all neurons in the network through repeated LTP [28]. The recruited neurons then acted as new seeds for the network growth, and recruited their own targets just like the training neurons did. This iterated as the trials went on, and the synaptic network grew through the neuron-by-neuron recruitment process.

In the model, network grew gradually as neurons were added to the network (Fig 4F). The total time of burst propagation in the network gradually increased with the number of training trials. The network topology was visualized with the Kamada-Kawai algorithm [47], which lays out the neurons in the network in a two-dimensional space such that the Euclidian distance between any pair of the neurons roughly matches the minimum number of the strong connections needed to connect the pair in the network, with the priority of matching given to the directly connected pairs. The resulting plot revealed a chain network structure with the training neurons at one end and the growth edge at the other. The length of the chain grew with the number of training trials. Added neurons were initially immature and had less tight burst compared to the neurons already in the network. With time and reliable activations, the added neurons matured and developed tight bursts. Thus, we always had immature neurons at the growth edge of the network. This can be seen in Fig 4F (bottom row), which shows that neurons bursting at the end of the activity propagation in the chain have larger first interspike intervals than those that burst early.

During the growth, some immature pool neurons were not recruited due to the competitive process of the recruitment. However, these neurons still matured with time, although the maturation rate was much less compared to that of the recruited neurons, which bursted reliably at each trial. As they matured, the non-recruited neurons became less spontaneously active, which further reduced the chances that they could be recruited. Eventually they became completely mature and lacked spontaneous activity, leaving them silent and outside of the synaptic chain network. In our simulations, we introduced neuronal turnover to eliminate these silent pool neurons. If a maturing pool neuron spiked in less than 80 trials in the past 4000 trials, it was replaced by a new immature neuron (S2 Fig). This turnover process mimics the turnover of newborn HVC_RA_ neurons observed in juvenile zebra finches [40]. Since the silent neurons were replaced in our simulations, the network consisted of immature pool neurons with varying degrees of “maturity” that depended on their “birth times”, defined as the trial numbers at which the neurons were introduced as new immature neurons. The neurons in the growing network were all mature neurons, except those newly recruited and attached to the growth edge. In our simulations, the fraction of the pool neurons of the total number of neurons decreased as more neurons were incorporated into the network. The fraction started from 1 and gradually approached around 0.6.

**Fig 5:**
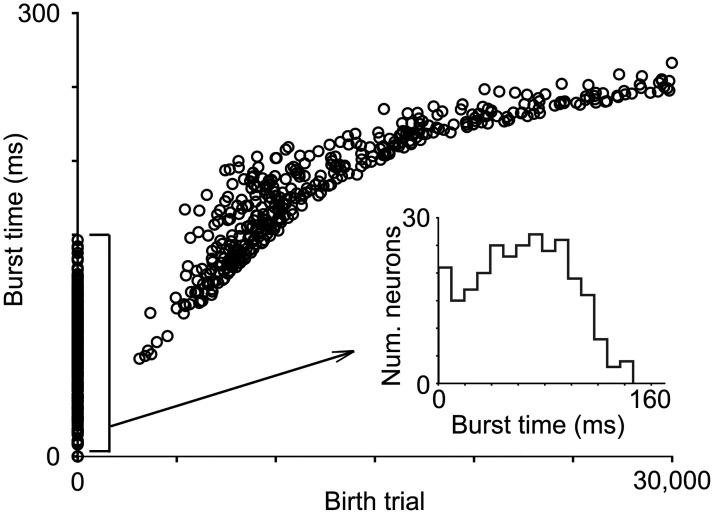
“Birth time” and burst time. The “birth time” is the trial number at which a new immature HVC_RA_ neuron is introduced to replace a non-recruited silent neuron. The birth time is positively correlated to the burst time of the neuron in the network. The points at trial 1 are the neurons that existed at the start of the wiring process. The insert plots the bursting time histogram for this population. The replacements starts at trial 4000 in the model. Soon after the replacement starts, the new immature neurons are more likely to get recruited than the resident neurons that have been maturing since their birth times. This can be seen in the insert: Soon after the replacements have started, the number of neurons that existed at trial 1 and recruited to the network trails off.

A consequence of neuronal turnover in our model is that the birth time of a recruited HVC_RA_ neuron positively correlates with the time at which it bursts in the network (Fig 5). This is because a new immature neuron has a higher spontaneous activity rate than the “resident” pool neurons that have been maturing since they are introduced to the pool. At each trial, more active pool neurons have higher chance of spiking right after the recruiting neurons, hence are more likely to get recruited. In our simulations, this positive correlation was apparent after the replacements started at trial 4000.

In our model, the growth of the network slowed down as the chain grew in length. There are two contributing factors for this phenomenon. One is that the fraction of the pool neurons decreased as the chain grew, hence less number of pool neurons were available to fire right after the recruiting neurons bursted at each trial. The other is that in our simulations each trial was limited to 500 ms, and the activation of the network was anywhere from 100 ms to 400 ms after the trial onset. As the network grew longer and the propagation of the bursts took more than 100 ms, some trials with late activations ended before the bursts could propagate all the way to the growth edge, and these trials had no recruiting events.

### Axonal conduction velocity and network topology

**Fig 6:**
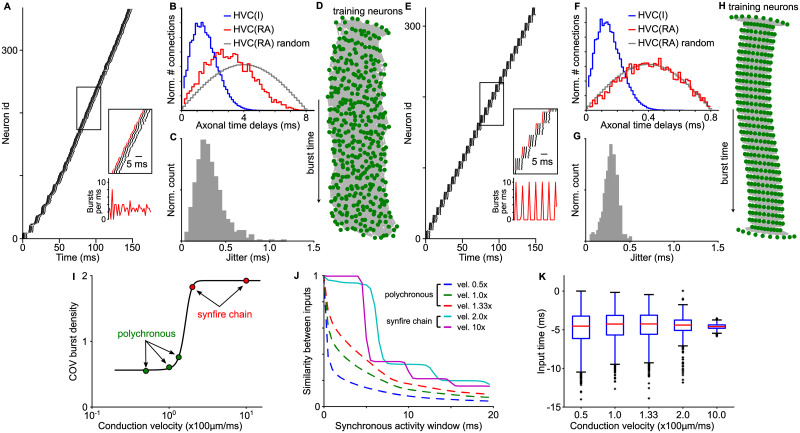
Axonal conduction velocity shapes network topology. A-D: Results for a network with conduction velocity 100 *μ*m/ms, which corresponds to the realistic axonal delays in HVC. A: Raster plot of the first 150 ms of dynamics shows continuous coverage of burst onset times. B: Axonal time delay distributions for the HVC_RA_ to HVC_INT_ connections (blue), and the HVC_RA_ to HVC_RA_ connections that emerged during the wiring process (red). As a reference, the delay distribution for randomly connected HVC_RA_ neurons is also shown (grey). The emerged connections show decrease in the number of long delay connections compared to the random connections. C: Jitter in burst onset times of a grown network. D: Network topology. Green dots are HVC_RA_ neurons, and the gray lines are the connections. Neurons on top are the training neurons. The arrow indicate the direction or burst propagation. Only neurons with burst onset times within the first 150 ms are shown. The network has no apparent grouping of neurons. E-G: Results for a network with 10x faster conduction velocity 1000 *μ*m/ms, which leads to near zero axonal delays. E: The network dynamics has prominent synchronous oscillatory activity. F: No bias towards shorter delay connections is observed in the grown network. G: The timing precision is in sub-millisecond range. H: Network topology reveals groups of neurons with similar input and output connections, i.e. synfire chain layers. I: The coefficient of variation of the burst onset density shows transition from continuous to discrete activity pattern. J: Similarity of inputs for neurons bursting within synchronous activity window has plateaus for synfire chain networks and is smooth for continuous networks. K: Distributions of excitatory input times relative to burst onset time of the postsynaptic neurons for different conduction velocities. The inputs arrive at the postsynaptic neurons synchronously in all networks wired with different conduction velocities.

In our model, the axonal conduction velocity controlled the axonal time delays between neurons. With the conduction velocity set to 100 *μ*m/ms, which created the realistic axonal time delays between HVC_RA_ neurons [21], the emerged network showed continuous dynamics and nearly uniform temporal distribution of burst onset times (Fig 6A). Established connections between HVC_RA_ neurons (Fig 6B) were biased towards short delay connections, but were on average longer than the connections to HVC_INT_ neurons that were set with the distance based probabilistic rule. The network was temporally precise with a sub-millisecond jitter in burst onset times (Fig 6C). Plot of the network topology with the Kamada-Kawai algorithm [47] did not reveal any grouping structure (Fig 6D). These are the characteristics of the polychronous chain network proposed as the connectivity of HVC_RA_ neurons within HVC in a recent study [21].

When we repeated the growth with a 10 times faster conduction velocity (1000 *μ*m/ms), the emerged network showed a strongly synchronous activity pattern (Fig 6E). The distribution of axonal delays between HVC_RA_ neurons in the formed network was similar to the delay distribution of randomly connected HVC_RA_ neurons (Fig 6F). The network was also temporally precise with the jitter level similar to the polychronous chain network (Fig 6G). The network topology was highly structured, showing groups of neurons with similar input and output connections. In other words, the grown network had a synfire chain topology with prominent oscillatory activity coming from the identical chain layers of neurons.

We systematically varied conduction velocity from 0.5 to 10 times of the original value, and observed a sharp transition in the burst density oscillations at 1.5 (Fig 6I). Networks with the velocity smaller than this value had a flat burst density, while networks with velocity exceeding this value showed prominent oscillations. We quantified the network structure using the similarity of input connections for the neurons bursting simultaneously in a time window of variable size (Fig 6J). The networks with prominent oscillations in burst density (vel. 2 and 10 times) showed a stair-like decay in the similarity of inputs, which is expected for the synfire chain topology with defined groups and all-to-all connections from the neurons in one group to the next; whereas the networks with weak activity oscillations (vel. 0.5, 1 and 1.33 times) had a smooth decreasing curve, which is expected for the polychronous chain networks with no definable groups. All grown networks, regardless synfire chains or polychronous chains, possessed a property of nearly synchronous excitatory inputs to the postsynaptic neurons (Fig 6K).

**Fig 7:**
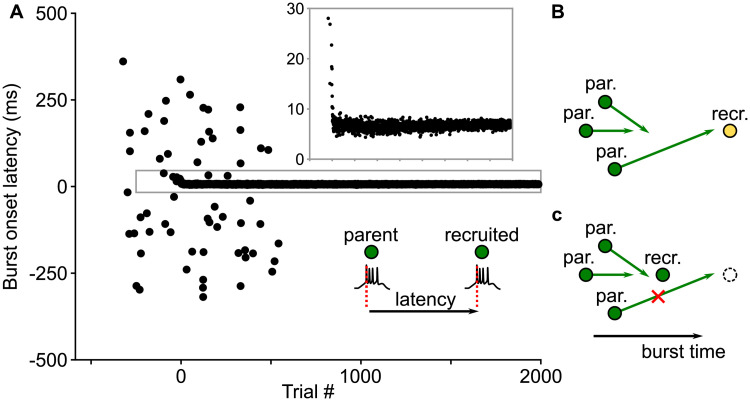
Decrease in the burst onset latency of the recruited neurons leads to pruning of long delay connections. A: Burst onset latency between the parent and the recruited neurons decreases during recruitment. Early in the recruitment, the recruited neurons are immature can still spontaneously fire, hence the burst onset latencies are scattered in a large range. This scatter disappears in later trials as the neurons become mature. The boxed area is scaled to show more clearly the burst events of the recruited neurons driven by the parents in all trials. Steady decrease of the burst onset latencies with the training trials is evident. B-C: Mechanism for pruning long delay connections. B: A neuron being recruited initially spikes at a large latency, which allows long delay connections to emerge. C: After the recruitment, the neuron spikes at a shorter latency, which makes the long delay connections to arrive late and be pruned via LTD.

To understand how the conduction velocity influences the network topology, we examined the case of slow conduction velocity, for which the potential connections between neurons have a wide range of axonal delays. We monitored the burst onset latency of the recruited neurons relative to their presynaptic neurons (the parents) (Fig 7A). In the beginning of the recruitment, connections to the recruited neurons were still weak and these neurons had a large range of burst onset latency. This permitted connections with a large range of delays to target the recruited neurons via LTP (Fig 7B). Subsequently, however, the burst onset latency was gradually decreasing due to strengthening of the connections from the parent neurons (Fig 7A, inset). This resulted in pruning of some of the inputs with long axonal delays via LTD (Fig 7C). Therefore, the grown network had a prominent bias towards forming short delay connections while keeping a few long delay connections, characteristic of the delay distribution for the polychronous chain topology. In contrast, when the conduction velocity is high, all possible connections have short delays, and there is no bias towards short distance connections. In this case, the synfire chain topology emerged.

### The role of inhibition in network growth

Inhibition should play an important role in the network growth since it impacts the spontaneous activity of immature neurons. Due to the randomness of the connections between HVC_RA_ neurons and HVC_INT_ neurons, feedback inhibition to individual HVC_RA_ neurons is inhomogeneous in time. To see if this affects which neurons get recruited into the network, we tracked the inhibitory conductance of all HVC_RA_ neurons in the network. We considered a simulation with the default conduction velocity (100 *μ*m/ms), and switched off the replacement of the silent non-recruited neurons to allow a direct comparison between the recruited and the non-recruited neurons. The grown network after 30,000 trials contained 306 recruited and 1,684 non-recruited HVC_RA_ neurons.

**Fig 8:**
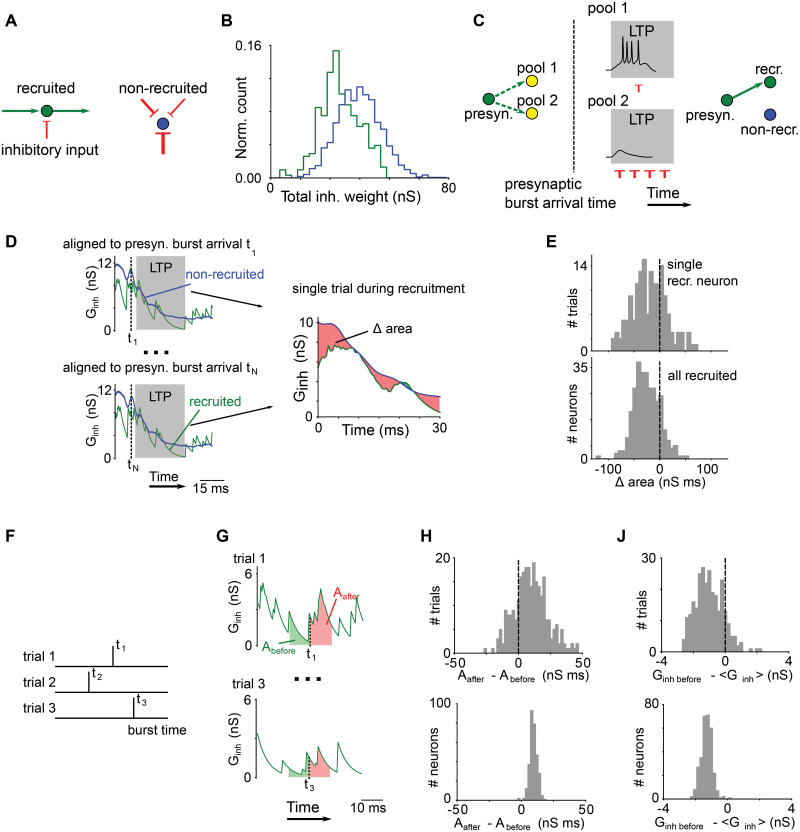
The role of inhibition in the network growth. A-B: Comparison of inhibitory weights onto the recruited and the non-recruited neurons. A: A recruited neuron (green circles) receives strong excitation (green arrow) and weak inhibition (red arrow). A non-recruited neuron (blue circles) receives larger inhibitory inputs. B: Distribution of the total inhibition, computed as the sum of all inhibitory input conductance to the neuron, shows stronger inhibition onto the non-recruited HVC_RA_ neurons (blue) compared to the recruited neurons (green), *p* < 10^−39^, Wilcoxon rank-sum test. C-E: Comparison of the temporal profile of inhibitory conductance aligned to the presynaptic neuron bursts during the network growth. C: Illustration of how inhibition determines which HVC_RA_ neurons get recruited into the network. Left: the presynaptic HVC_RA_ neuron (green circle) may form connections to two pool HVC_RA_ neurons (yellow circles) during the network growth. Middle: When aligned to the presynaptic burst arrival time, pool neuron 1 receives weak inhibition (red arrows) in the LTP window, defined as the time interval in which bursting of the pool neuron leads to a significant strengthening of the connection from the presynaptic neuron. Due to weak inhibition, pool neuron 1 can spike. Pool neuron 2 receives strong inhibition in the LTP window and its spiking activity is suppressed. Right: Eventually pool neuron 1 gets recruited into the network, while pool neuron 2 remains not recruited. D: Left: For each recruited neuron during the network growth, the temporal profile of inhibitory conductance is aligned to the burst arrival times of all presynaptic parent neurons (green). Note that these arrival times are synchronous but have small jitters across different presynaptic neurons. At each trial, the inhibitory conductance in the LTP window is averaged across all parent neurons during the network growth. For comparison, we use an averaged inhibitory conductance of all non-recruited HVC_RA_ neurons extracted in the same time intervals. Right: The inhibitory conductance in the LTP window averaged for all presynaptic burst alignments is compared between the recruited (green) and the non-recruited (blue) neurons using the area Δ under the conductance curve. E: Top: Δ for a single recruited neuron during the network growth until the neuron is recruited. Bottom: Δ for all recruited neurons. For each recruited neuron, we use the median Δ across all trials until the neuron recruitment. Distribution has a negative shift, meaning that the recruited neurons receive smaller inhibitory conductance in the LTP window during the recruitment, *p* < 10^−34^, Wilcoxon signed-rank test. F-H: Comparison of the temporal profile of inhibitory conductance aligned to the postsynaptic neurons during the network growth. F: Burst times of a neuron being recruited at different trials. G: The inhibitory conductance profile is aligned to the burst onset times of the recruited neurons. Difference in inhibitory conductance in time windows 10 ms after and 10 ms before the burst is calculated using the area under the conductance curve, *A*_before_ and *A*_after_. (**h**) Top: *A*_before_ − *A*_after_ for a single recruited neuron during the network growth until the neuron is recruited. Bottom: *A*_before_ − *A*_after_ for all recruited neurons. For each recruited neuron, we used the median of the difference across all trials until the neuron recruitment. The distribution has a positive shift, meaning that the recruited neurons receive stronger inhibition after the burst, *p* < 10^−51^, Wilcoxon signed-rank test. J: Top: The difference in inhibitory conductance 10 ms before the burst and the mean inhibitory conductance in the trial for a single recruited neuron during the network growth until the neuron is recruited. Bottom: The distribution of the difference for all recruited neurons. For each recruited neuron, we used the median of the differences in all trials until the neuron recruitment. The distribution has a negative shift, meaning that the recruited neurons receive smaller inhibition before the burst compared to the average inhibition during the entire trial, *p* < 10^−51^, Wilcoxon signed-rank test.

We observed that in this network, the averaged inhibitory synaptic weight to single non-recruited neurons (1515 ± 4 pS, mean ± s.e.m.) were larger than the average to single recruited neurons (1430 ± 9 pS, *p* < 10^−17^, Wilcoxon rank-sum test). Additionally, the number of inhibitory connections to single non-recruited neurons (32.2 ± 0.1) was also larger than the number to single recruited neurons (29.7 ± 0.2, *p* < 10^−22^, Wilcoxon rank-sum test). It resulted in an increased total inhibition (computed as a sum of all inhibitory conductance to a neuron) to the non-recruited neurons (48.7 ± 0.2 nS) compared to the total inhibition to the recruited neurons (42.4 ± 0.4 nS, *P* < 10^−39^, Wilcoxon rank-sum test; Fig 8A and 8B).

Timing of the inhibitory inputs is also an important factor in determining which neurons get recruited. This is illustrated with a simple example (Fig 8C). Consider a neuron at the growth edge of the network forming connections to two pool neurons. After a burst of the network neuron arrives at the pool neurons, the connections are strengthened if the pool neurons fire in the LTP window of the BTDP synaptic rule (between 2 ms and 32 ms after the presynaptic burst arrival). In this example pool neuron 1 receives less inhibition than pool neuron 2 in the LTP window, hence pool neuron 1 is more likely to fire than pool neuron 2; therefore, pool neuron 1 is more likely to get recruited.

To quantify the effect of inhibition in the LTP window in our simulations, we compared the temporal profiles of inhibitory conductance of the recruited and the non-recruited HVC_RA_ neurons during the network growth. We aligned the profile of a recruited neuron to the burst arrival time of the presynaptic parent neurons, and compared the profile in the LTP window to the averaged inhibitory conductance profile of the non-recruited neurons extracted in the same time interval (Fig 8D and 8E). We took the difference of the inhibitory conductance at each time point and summed over the time window to arrive at the total area of the difference Δ. We found that over multiple trials, Δ for a single recruited neuron tended to be negative, indicating that the neuron received less inhibition in the LTP time window than the non-recruited neurons (Fig 8E). For the population, the median of the distribution for the averaged Δ for the recruited neurons was significantly negative (*p* < 10^−34^, Wilcoxon signed-rank test; Fig 8E). This observation shows that neurons that receive less inhibition from the parent neurons are preferentially recruited into the growth edge of the network. For fully grown network, because the excitatory connections are much stronger than during the recruitments, the inhibition is not able to suppress the activations of the postsynaptic neurons. Nevertheless, we see that Δ is still significantly negative (*p* < 10^−26^, Wilcoxon signed-rank test; S3 Fig).

We next aligned the inhibitory conductance profiles of the recruited neurons to their own bursts during the network growth. We compared the inhibitory conductances of a recruited neuron in two time windows: from 10 ms before the burst to the burst, and from the burst to 10 ms after the burst (Fig 8F and 8G). The sum of inhibitory conductance in the before-window is denoted as *A*_before_, and that in the after-window as *A*_after_. The difference *A*_before_ − *A*_after_ for a single recruited neuron over multiple trials tended to be positive (Fig 8H), indicating that the recruited neuron received more inhibition after its own burst than before. As a population, the distribution of the median differences for all recruited neurons had a mean that was significantly positive (*p* < 10^−51^, Wilcoxon signed-rank test; Fig 8H). We attribute this observation to the self-inhibition of the neurons due to the prevalence of local connections between HVC_RA_ neurons and HVC_INT_ neurons. By bursting, HVC_RA_ neuron activated a subset of nearby interneurons, which in turn provided a feedback inhibition. In the fully grown network, this effect is much smaller but still significant (*p* < 10^−5^, Wilcoxon signed-rank test; S3 Fig). The effect is small due to the high network driven activity of HVC_INT_ neuron population.

During the network growth, the inhibitory conductance on the recruited neurons 10 ms before the burst was smaller than the mean inhibitory conductance computed over the trials (*p* < 10^−51^, Wilcoxon signed-rank test; Fig 8J). This further supports that HVC_RA_ neurons requires less inhibition on average to be recruited. Since the initial excitatory inputs to HVC_RA_ neurons are weak, the recruitment favors HVC_RA_ neurons that receive less inhibition to ensure that they can be activated by the parent neurons at the growth edge. In the fully grown network, the inhibitory conductance 10 ms before bursts was significantly larger than the averaged inhibitory conductance during the trials (*p* < 10^−50^, Wilcoxon signed-rank test; S3 Fig). This is simply because the network activity was after a period of spontaneous activity in each trial, and the firing rates of the inhibitory neurons were much higher during the network activity than during spontaneous activity. Note that the network activity started anywhere between 100 to 400 ms in each trial in our simulations.

### Experimental evidence linking maturity of HVC_RA_ neurons and sequence growth

Experimentally, Okubo et al showed that the length of sequential activity of HVC_RA_ neurons grows during vocal development in zebra finches [17]. To see whether immature neurons are involved in the sequence growth, we reanalyzed the publicly available Okubo dataset, which contains extracellular recordings of neurons in HVC of juvenile zebra finches [17, 48]. The dataset is organized into four stages of song development [17]: subsong, protosyllable song, multi-syllable song, and motif song.

**Fig 9:**
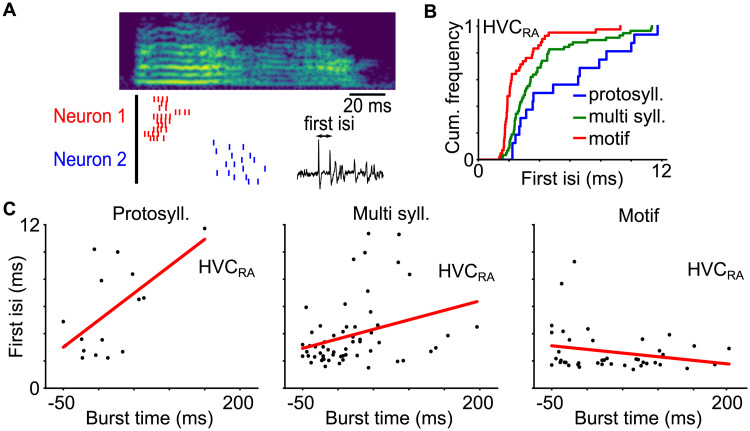
Burst tightness of HVC_RA_ neurons as quantified with the first interspike interval at different stages of song development. A: Example of spike patterns of two HVC_RA_ neurons in the protosyllable stage aligned to a syllable onset. B: Cumulative distributions of the first interspike intervals of HVC_RA_ neurons. The first interspike intervals become progressively smaller on average as the song develops (multi-syllable versus protosyllable, *p* = 0.023, one-sided Wilcoxon rank sum test; motif versus multi-syllable, *p* < 0.0001, one-sided Wilcoxon rank sum test). C: The first interspike intervals of HVC_RA_ neurons as a function of the burst times at the protosyllable, the multi-syllable and the motif stages. The red line is the linear least square fit. The slope is significantly positive for the protosyllable stage (*p* = 0.012, two-tailed t-test). The trend is weaker but significant for the multi-syllable stage (*p* = 0.017, two-tailed t-test), and is not significant for the motif stage (*p* = 0.14, two-tailed t-test).

HVC_RA_ neurons in adult zebra finches produce highly stereotyped bursts of 4—5 spikes lasting approximately 6 ms [8]. Experiments and computational models suggest that such a burst is driven by dendritic calcium spike [9, 22]. Since immature neurons typically do not have fully developed dendritic trees [31, 49], immature HVC_RA_ neurons may not be able to generate brief, high frequency bursts. Indeed, spike patterns of projection neurons during song development varied significantly in the number of spikes produced per burst and in the burst duration [17]. We therefore assumed that burst tightness is an indicator for HVC_RA_ neuron maturity. Specifically, we defined burst tightness as the first interspike interval in the burst (Fig 9A). The first spike interspike interval is simple to measure and is not affected by the variations in the durations and the numbers of spikes in the bursts. We observed that bursts in the HVC_RA_ neuron population in the data gradually tightened as the song progressed through the protosyllable, multi-syllable and motif stages (Fig 9B, multi-syllable versus protosyllable, *p* = 0.023, one-sided Wilcoxon rank sum test; motif versus multi-syllable, *p* < 0.0001, one-sided Wilcoxon rank sum test), supporting that burst tightness is positively linked to song development and presumably to HVC_RA_ neuron maturation.

We next looked at the burst tightness of the HVC_RA_ neurons that were locked to syllables, *i.e.* those tended to burst at fixed latencies relative to the syllable onsets (Fig 9C). In the protosyllable stage, the first spike interval significantly increased with the burst latency (*p* = 0.012, two-tailed t-test), suggesting that bursts are tighter for neurons bursting at the start of the syllables than those at the end. Thus, the maturity of HVC_RA_ neurons are heterogeneous in this stage, and immature neurons tend to burst towards the end of the syllables. This trend was less pronounced but still significant in the multi-syllable stage (*p* = 0.017, two-tailed t-test). It disappeared in the motif stage (*p* = 0.14, two-tailed t-test).

Our analysis provides evidence that the maturity of HVC_RA_ neurons is correlated with their burst timings during the early stages of song development, and that immature neurons are preferentially added to the end of the growing sequence in HVC.

## Discussion

In an adult zebra finch, HVC_RA_ neurons burst sequentially with a millisecond precision during singing [8]. Electrophysiological [10] and calcium imaging [11] studies showed that the sequence is continuous, supporting the idea that such sequential bursts are generated within HVC through a feedforward synaptic chain network [9, 18, 22]. Previous models suggested that such a network can be wired by recruiting neurons group by group through synaptic plasticity and spontaneous activity, resulting in growth of sequence during the wiring process [28, 29]. This prediction is in agreement with an experiment that recorded projection neurons in HVC of juvenile zebra finches [17]. Our reanalysis of this experimental data [48] suggested that HVC_RA_ neurons at the growth edge have the characteristics of immature neurons. We therefore further extended the model to include the maturation dynamics of HVC_RA_ neurons. Moreover, we included more biologically realistic features that lacked in previous models, including explicit modeling of HVC_INT_ neurons, spatial distributions of HVC neurons, and realistic axonal delays in HVC [21]. We showed that immature neurons, which are more excitable hence have higher spontaneous activity rates compared to mature neurons, are preferentially recruited at the growth edge. The inclusion of the axonal delays leads to a long polychronous chain network, a structure favored by a recent analysis of HVC network and dynamics [21]. In contrast, neglecting axonal delays leads to synfire chains [37, 50], previously thought to be the topology of the HVC network [22, 28, 29]. Explicit modeling of HVC_INT_ also predicts that the wiring process favors a path of less inhibition, such that neurons that are recruited receive less forward inhibition from the recruiting neurons, highlighting the importance of inhibition in HVC [19]. Our model also reproduces the observation that HVC_RA_ neurons connect to more distal HVC_RA_ neurons, unlike their tendency to connect to nearby HVC_INT_ neurons [20].

Inclusion of immature neurons has an important effect on the growth process of synaptic chain networks. In the model, spontaneous activity plays a critical role. The distinction between immature and mature neurons allows different levels of spontaneous activity in these two populations. Immature neurons are more spontaneously active due to higher intrinsic excitability, and they are the targets of recruitments by the neurons at the growth edge. In contrast, mature neurons in the network are not spontaneously active, hence are not targets of recruitments. This allows continued growth of the network, as long as there is a supply of immature neurons in the pool. This was not the case in the previous models, in which there was a single neuron population [22, 28, 29]. There, all neurons had similar level of spontaneous activity and consequently, the chain growth usually stopped by the formation of loops after neurons already in the chain were recruited. We have confirmed that loops emerge in our model as well when using a single population of mature and spontaneously active HVC_RA_ neurons (S4 Fig).

During development, immature neurons in many neural circuits across multiple species go through a period of depolarizing inhibition before switching to hyperpolarizing inhibition, which is caused by an elevated GABA reversal potential on immature neurons [51]. Our computational experiments with developmental switch in GABA resulted in the emergence of numerous connections between nearby HVC_RA_ neurons [52]. This was because the dense local connectivity between HVC_RA_ and HVC_INT_ neurons promotes recruitment of nearby immature neurons through depolarizing local inhibition. Experimentally, local connections between HVC_RA_ neurons are sparse [20]. We therefore assumed that the emergence of connectivity between HVC_RA_ neurons happens at the time when GABA exerts a hyperpolarizing response on immature neurons. This assumption needs to be tested in future studies with intracellular recordings of HVC_RA_ neurons during development.

In our model, maturation of newborn HVC_RA_ neurons is age and activity driven. The passage of time alone is enough for the neuron to mature, but more reliable activations after the recruitment into the network accelerates the maturation. This acceleration prevents the recruited neurons in the growing network from spontaneous activations, and hence protect the network from forming loops. This maturation dynamics is inspired by the observation that adult-born neurons in rodent hippocampus mature faster with enhanced activity and mature more slowly with reduced activity [53]. The exact value of the activity-driven maturation time scale is not important, as long as it is much smaller than the maturation time scale with the age and spontaneous activity alone. Neurons that become mature but not recruited into the network become silent, and they are replaced by new immature neurons. This turnover ensures that there is a fresh supply of immature neurons for the chain growth. The rate of replacement also controls the number of available targets for the growth, which is important for forming convergent inputs to the targets during the recruitment process. If the number of targets is too large, the recruiting neurons can connect to divergent targets, and the resulting network is not capable of producing precise timing.

A consequence of the chain growth and the turnover is that the burst times of neurons in the chain network is positively correlated with the order of their introductions. In other words, birth order determines burst timing. Since neurons are added to the growth edge, newly added HVC_RA_ neurons, if recruited, should burst at the time point of the growth edge. Hence the growth of the chain network necessarily requires late burst times for late born neurons. Additionally, newly born immature neurons are more spontaneously active compared to the resident maturing neurons, and they are better positioned to be recruited to the growth edge at the time of their arrivals in HVC. This prediction of our model can be tested by labeling cohorts of newborn neurons using viral strategy in juvenile [25] and recording their burst timings in adulthood using calcium imaging [11].

Our simulations started with all neurons immature except the training neurons. We judged the silent neurons that should be replaced by new immature neurons based on their spontaneous activity in the past 4000 trials. Consequently, the neuronal turnover happened only after the network had grown for some time (Fig 5). Once the turnover started, the positive correlation between the birth time and the burst time became apparent. In real HVC, it is more likely that when the wiring process starts, there are already a mixture of newly born neurons, maturing neurons, and fully matured neurons. We therefore expect that the correlation should apply to all neurons in the grown network in real HVC.

Addition and turnover of HVC_RA_ neurons post hatch has been observed for over 30 years [23, 54], but the significance of this process for birdsong development remains unclear [26, 30]. In juvenile zebra finch, deprivation of auditory inputs by deafening before song learning [55] and the inability to learn tutor song due to peripheral nerve injury [56] did not impact the addition of newly born HVC_RA_ neurons. These observations are consistent with our view that the addition of newly born HVC_RA_ neurons mainly contributes to the self-organized wiring process of the synaptic chain network in HVC, which should not depend on auditory inputs or learning specific tutor songs.

Synfire chain is a popular feedforward model for generating precise and stable sequential activity of neurons [37, 50, 57]. Several computational models have explored the formation of synfire chains. Successful models that can grew long sequences used a combination of the STDP rule and additional synaptic plasticity mechanisms to constrain the connectivity. With the STDP rule and heterosynaptic plasticity rules that limited the total incoming and outgoing synaptic weights for each neuron, Fiete et al [58] showed formation of synfire chain loops with length distributed according to a power law. Short loops were more numerous than long loops. However, to form groups of neurons that fire at the same time as observed in HVC, the model needed to introduce additional correlated inputs that defined coherent groups before the chain formation. Jun and Jin [28] showed that synfire chain forms with the STDP rule and additional synaptic plasticity rules that constrained the number of strong output connections. The model was able to show the gradual growth of synfire chains through group-by-group recruitment of HVC_RA_ neurons. The process ended with the formation of a loop, with the length following a Gaussian distribution [29].

Our study builds upon the gradual recruitment model [28, 29] and uses similar synaptic plasticity rules. However, our model introduces several realistic features that none of the previous models had, including explicit modeling of HVC_INT_ neurons; spatial distributions of neurons and realistic axonal time delays recently measured in HVC [21]; and, most importantly, newly born HVC_RA_ neurons and their maturation dynamics. These lead to novel insights, as discussed earlier. Additionally, no loops form in our model, unlike all previous models. Under realistic axonal time delays, we show that a polychronous chain network rather than a synfire chain network emerges after the training. The network possesses a sub-millisecond precision, and importantly, the bursts of the neurons cover the time almost uniformly with no preferred time points. Using connections with fast conduction velocity, we can recover the synfire chain topology. The grown synfire chain network has similar sub-millisecond level of precision, but its burst density shows prominent oscillations with some time points with more bursts than others. By changing the axonal conduction velocity between HVC_RA_ neurons, we can grow either synfire chain or polychronous chain networks. In the polychronous chains, neurons are driven by almost synchronous inputs despite of the distributed presynaptic spike times due to the delays. This is similar to a previous study in which approximately 70 ms long polychronous sequences with an average size around 20 neurons emerged and disappeared in a recurrent network with the STDP rule for synaptic plasticity [38]. However, in our case the incorporation of additional synaptic plasticity rules produce stable sequences that span hundreds of milliseconds and contain hundreds of neurons. Thus, we show that long polychronous neuronal sequence can emerge from a combination of the STDP and additional synaptic plasticity rules.

Our growth mechanism is robust with respect to the changes in the model parameter values. The use of different strength of inhibitory connections (varied between *G*_*ie*_ = 0.015 *mS*/*cm*^2^ and *G*_*ie*_ = 0.060 *mS*/*cm*^2^), different number of efferent supersynaptic connections (*N*_*s*_ = 10 and *N*_*s*_ = 20), and different maximal strength of excitatory connections between HVC_RA_ neurons (between *G*_*max*_ = 1.5 *nS* and *G*_*max*_ = 4 *nS*) lead to the emergence of precisely timed neural sequences [52]. Thus our modeling results do not rely on fine-tuning of the model parameters.

Our reanalysis of the data that recorded HVC neurons in juveniles [17, 48] showed that the burst tightness of projection neurons decreases with the burst timing during the sequence growth in the protosyllable stage. This difference disappears in later stages of song development. We interpreted decreased tightness of bursts as a reflection of immature intrinsic bursting mechanism. An alternative possibility is that the burst tightness is a network phenomenon. It is possible that neurons that burst earlier in the sequence are better connected and get stronger inputs, leading to tighter bursts, whereas those that burst later are still in process of getting incorporated and hence are loosely connected. Another possibility is that feedback inhibition controls the burst tightness [59]. However, there is some evidence in the data that supports the intrinsic mechanism. We found one HVC_RA_ neuron in the subsong stage that was not locked to a syllable but still showed tight bursts usually observed in the motif stage (S5 Fig). Since the network is unlikely formed in this stage, this observation favors intrinsic mechanism for burst tightness. It is also possible to access the network effect on the burst tightness by comparing the spike patterns of syllable-locked neurons during and outside of singing of the syllables. However, due to the limited number of HVC_RA_ neurons recorded in the subsong stage and the protosyllable stage in the experiments [17, 48], which were not designed to address these questions, we could not gather more evidence to be certain about the network effects. Future experiments with more data on HVC_RA_ neurons in early stages of song development, perhaps also including intracellular recordings *in vivo* and in slices, should be able to address whether burst tightness is intrinsically controlled.

Intermittent activations of a set of training neurons and spontaneous activity of immature neurons are two of the most important ingredients of the network growth process in our model. As in the previous models of chain growth, the activations of the training neurons create the recruiting events in the wiring process [28, 29]. The thalamic nucleus Uvaeformis (UVA) and the cortical nucleus interfacialis of the nidopallium (NIF), an area that is highly selective to the bird’s own song [60], are two major sources of inputs to HVC [60–64]. As early as 20 days post hatch, UVA and NIF have already established projections to HVC [65, 66]. It is conceivable that the HVC_RA_ neurons innervated by UVA and/or NIF around this age or even earlier are the ones that act as the training set for the network growth. Indeed, UVA neurons do burst consistently before the onsets of syllables [63], as do NIF neurons [64]. A recent study provides evidence that NIF neurons are more likely candidates for providing the inputs to the HVC training neurons [64]. Spontaneous bursts in UVA and NIF can reliably activate HVC neurons [62]. We speculate that the chain growth starts as soon as the UVA and/or NIF projections are established to HVC_RA_ neurons, and spontaneous bursting appears in UVA, NIF, and HVC. The wiring process most likely goes on well into the protosyllable stage, since we see from the reanalysis of the data [17] evidence that the maturity of HVC_RA_ neurons is correlated with the timing of their bursts relative to the syllable onsets in this stage [17] (Fig 9), as expected from our model (Fig 4). The protosyllable stage is when definable durations of syllables emerge while the acoustic features of the syllables are not yet learned [17]. The establishment of synaptic chain network in this stage should provide the timing substrate that is required for establishing the specific projections from HVC to RA to learn the specific syllables through reinforcement learning [12–15]. Taken together, we propose that the wiring process of the synaptic chain network in HVC spans a time before the subsong stage to the protosyllable stage (<20 to ∼58 dph).

In our model, the wiring process consists of trials spanning 500 ms. This time span is an arbitrary choice of the model. In reality, each trial should corresponds to a bursting event in UVA and/or NIF that activates HVC_RA_ neurons. The lengths of the trials then correspond to the inter-burst intervals of these events. These events might be most reliable during the singing attempts in the subsong and the protosyllable stages. However, they could also occur during sleep [8] and in other states of juvenile zebra finches. Our model shows that the maximum length of the chain is limited by the durations of the trials. It would be interesting to see whether the inter-burst intervals in UVA and/or NIF in juvenile zebra finch might dictate the durations of the syllables that emerge at the protosyllable stage.

We used synaptic plasticity rules based on the timing of burst onsets (the BTDP rule). This simple rule sidesteps the complex interactions of multiple spikes within the bursting pre- and post-synaptic neurons [67], and is guided by the observation that in cortical neurons, the timings of the first spikes in bursts are most important for determining the timing-dependent LTP and LTD [68]. In addition, we apply a small 2 ms shift of the BTDP curve to the region of positive times, so that there is an LTD for synchronously bursting neurons. This prevents the emergence of connections between neurons that fire synchronously. Such a shift was used to stabilize weight distributions in random networks of spiking neurons in another modeling study [69]. Whether these rules apply to synaptic plasticity for HVC_RA_ neurons remains to be seen. To date, there is no systematic study of synaptic plasticity in HVC, and further experiments are needed.

In addition to sequence growth, extracellular recordings in juvenile zebra finches also revealed sequence splitting during the syllable development [17]. At the protosyllable stage, a majority of the projection neurons fired in a single protosequence. When several syllable types emerged from a common protosyllable, the corresponding protosequence split. While there were still neurons firing at all syllables with the same latencies relative to syllable onsets (“shared neurons”), more neurons fired specifically to a single syllable type. Gradually, the shared neurons disappeared. The authors proposed a model, according to which a protosequence grown from a common seed of synchronously activated neurons is split by dividing the seed into several groups activated at different times, and also by increasing local inhibition. In our study, the splitting does not happen during the network growth and we did not explore mechanisms for it to happen. Activation of seed neurons at different times and increase in inhibition may also induce protosequence splitting in our model.

In conclusion, we have shown that protracted addition of new neurons in HVC in juvenile helps to wire synaptic chain network through a self-organized process. Our model illustrates the possibility that birth order of neurons is important for constructing functional microcircuits in local brain areas.

## Materials and methods

### Juvenile zebra finch data analysis

We reanalyzed a previously reported data set of extracellular recordings in HVC of juvenile zebra finches [17, 48]. The data set contained recordings of projection neurons from 32 birds during the song development (44-112 dph). HVC_RA_ neurons exhibited sparse bursting activity. Following the procedure in Okubo et al [17], a burst was defined as a continuous group of spikes separated by intervals of 30 ms or less. To determine the burst tightness of a projection neuron, we estimated the median of the first interspike intervals of all the bursts produced by the neuron at a given song development stage (subsong, protosyllable, multi-syllable, and motif). To find the bursting time of the neurons locked to syllables, we followed the approach in Okubo et al [17].

### Network model

We distributed 2000 HVC_RA_ and 550 HVC_INT_ neurons over the 2D sphere of radius 260 *μ*m with no overlap. A neuron occupies a volume of a sphere with diameter 10*μm*. HVC_INT_ neurons were first placed evenly on the sphere using the Fibonacci lattice [70]. The distance between the nearest neighbors on sphere is approximately Δ*r*_*in*_ = 40 *μ*m, which matches the average distance between HVC_INT_ in real HVC (as estimated from the HVC volume and the number of interneurons). Then, they were randomly shifted along the sphere surface by a small amount: Δ*θ* = 0.0006Δ*r*_*in*_ and Δ*ϕ* = 0.0006Δ*r*_*in*_/*sin*(*θ*), where *θ* is the latitude of a neuron’s position on the sphere, *ϕ* is its longitude. HVC_RA_ neurons were placed randomly over the sphere, with the constraint that they do not overlap with other HVC_RA_ or HVC_INT_ neurons.

Connections between HVC_INT_ and HVC_RA_ neurons were placed probabilistically based on the distance between neurons along the sphere: $p_{RA\to I}\propto \exp\left(-d^{2}/\sigma_{RA\to I}^{2}\right)$ and $p_{I\to RA}\propto \exp\left(-d^{2}/\sigma_{I\to RA}^{2}\right)$, where *p*_*RA*→*I*_ is a probability for a given HVC_RA_ neuron to contact a given HVC_INT_ neuron, *p*_*I*→*RA*_ is a probability for a given HVC_INT_ neuron to contact a given HVC_RA_ neuron, *d* is a distance between given HVC_RA_ and HVC_INT_ neurons on the sphere, *σ*_*RA*→*I*_ = 130 *μm*, and *σ*_*I*→*RA*_ = 90 *μm*. Only a single connection between a pair of neurons was allowed. Parameter *σ*_*RA*→*I*_ was chosen to match the upper bound on the number of postsynaptic HVC_INT_ partners for an HVC_RA_ neuron [20, 71]. On average an HVC_RA_ neuron contacted 11.6% of HVC_INT_ neurons. HVC_INT_ neurons had a smaller spatial connectivity scale to influence nearby HVC_RA_ neurons. A single HVC_INT_ neuron contacted 5.8% of HVC_RA_ neurons. Conductance of the connections were sampled from uniform distributions on the intervals (0, *G*_*ei*_) for HVC_RA_ to HVC_INT_ connections and (0, *G*_*ie*_) for HVC_INT_ to HVC_RA_ connections, with *G*_*ei*_ = 0.4*mS*/*cm*^2^ and *G*_*ie*_ = 0.03*mS*/*cm*^2^. Axonal time delays for the connections were calculated by dividing the distance between neurons by the axonal conduction velocity. Normal conduction velocity was set to 100 *μ*m/ms, as observed in HVC [21]. Connections between HVC_RA_ neurons did not exist at the start of simulations.

A randomly selected set of 10 HVC_RA_ neurons were chosen as the training neurons that act as the starting seed for the network growth. The training neurons had the mature properties, while other HVC_RA_ neurons started as new immature neurons.

### Growth simulation

Network dynamics was run in trials of 500 ms duration with a time step 0.02 ms. In the beginning of each trial, the dynamical variables of neurons were reset to their resting values. At a random time between 100 ms and 400 ms in a trial, the training neurons were excited by a synchronous excitatory conductance kick of strength 300 nS, which made them burst. Simulations were run until the number of supersynaptic connections in the network remained constant for 10000 trials.

### Neuron model

For HVC_INT_ neuron we used a single compartment Hodgkin-Huxley model identical to the one described in [9]. For HVC_RA_ neuron we used a two-compartmental Hodgkin-Huxley model with soma and dendrite similar to the one in [9].

Parameters of sodium, potassium and leak currents of the soma of a mature HVC_RA_ are identical to those in [9]. Somatic compartment is additionally equipped with low-threshold potassium current *I*_*KLT*_ = *G*_*s*,*KLT*_ *l*(*V*_*s*_ − *E*_*K*_) with conductance *G*_*s*,*KLT*_ = 3.5 *mS*/*cm*^2^, potassium reversal potential *E*_*K*_ = −90 *mV* and gating variable *l*. Gating variable obeys the following dynamics: *τ*_*l*_*dl*/*dt* = *l*_∞_(*V*) − *l*, where *τ*_*l*_ = 10 ms, *l*_∞_(*V*) = 1/(1 + exp − (*V* + 40)/5). Parameters of the dendritic compartment of a mature HVC_RA_ are identical to [9], except for *τ*_*c*_ = 15 ms.

Immature HVC_RA_ neuron has elevated leak reversal potential *E*_*L*_ = −55 *mV* in both somatic and dendritic compartments. In addition, the calcium conductance in the dendritic compartment of immature HVC_RA_ were set to zero.

### Synapse model

Synaptic conductances on neurons were modeled according to the “kick-and-decay” dynamics [9]. Synaptic conductance of a neuron increases following a delivery of a spike to the synapse with conductance *G*: *g*_*syn*_ → *g*_*syn*_ + *G*. In between spike arrivals, synaptic conductance decays exponentially: *τ*_*syn*_ *dg*_*syn*_/*dt* = −*g*_*syn*_. We used the same values for synaptic decay time constants as in [9].

### Noise model and simulation

Noise in HVC_INT_ neurons was created using stochastic Poisson spike trains arriving at excitatory and inhibitory synapses, mimicking random synaptic activity, such that HVC_INT_ neurons spiked spontaneously with rate ∼ 10 Hz. Parameters of the Poisson spike trains were identical to [9]. Dynamics of HVC_INT_ neuron was solved using Dormand-Prince order 8 method [72].

Noise in HVC_RA_ neurons was implemented by injecting white noise current of amplitude 0.1 nA to soma and 0.2 nA to dendrite [21]. To account for white noise stimulus, HVC_RA_ model was treated as a system of stochastic differential equations and was solved with weak order 3 AN3D1 method [73].

### Maturation model

Maturation of HVC_RA_ neurons was modeled as a gradual increase of dendritic calcium conductance, and a gradual decrease in the somatic and dendritic leak reversal potential:

$$\begin{array}{c} \tau_{mat}\frac{dG_{Ca}}{dt}=G_{mat}-G_{Ca}, \end{array}$$

$$\begin{array}{c} \tau_{mat}\frac{dE_{L}}{dt}=E_{mat}-E_{L}, \end{array}$$

where *τ*_*mat*_ is the maturation time constant; *G*_*mat*_ = 55 *mS*/*cm*^2^ is the mature value of calcium conductance; and *E*_*mat*_ = −80 *mV* is the mature value of leak reversal potential. Values of *G*_*Ca*_ and *E*_*L*_ were updated at the end of each trial. Maturation rate of an HVC_RA_ neuron *τ*_*mat*_ depended on its activity history. If a neuron spiked in less than half of the trials in the past 1000 trials, it was treated as spontaneously spiking. Once a neuron spiked in more than half of the trials in the past 1000 trials, it was treated as reliably spiking. For a spontaneously spiking neuron, maturation time constant was set to *τ*_*mat*_ = 50,000 s. For a reliably spiking neuron, maturation time constant was set to a smaller value of *τ*_*mat*_ = 500 s.

### Neuronal turnover

Neuron was assigned as silent if it spiked in less than 80 trials in the past 4000 trials. Silent neurons were replaced at the end of each trial with new immature neurons. New immature neurons were placed randomly on the surface of the sphere representing HVC, avoiding overlaps with all HVC_RA_ and HVC_INT_ neurons.

### BTDP synaptic plasticity rule

To update weights between HVC_RA_ neurons, we used a BTDP rule based on the burst onset timing between the presynaptic and the postsynaptic neurons (Fig 4A). We defined a “burst” as a continuous group of spikes with duration 30 ms or less. Burst onset time was defined as the first spike in a burst. Each time a neuron produced a new burst, all afferent synapses onto the neuron and all efferent synapses are updated. For a pair of a presynaptic neuron *i* with burst onset time *t*_*i*_ and a postsynaptic neuron *j* with burst onset time *t*_*j*_, an additive LTP would occur for the synapse with weight *G*_*ij*_ if Δ*t* = *t*_*j*_ − *t*_*i*_ > *T*_0_:

$$\begin{array}{c} G_{ij}\to G_{ij}+\{\begin{array}{cc} A_{P}\left(\Delta t-T_{0}\right)/T_{P}, & \text{if}\;\Delta t<T_{0}+T_{P},\\ A_{P}\exp\left(-\left(\Delta t-T_{0}-T_{P}\right)/\tau_{P}\right), & \text{if}\;\Delta t\geq T_{0}+T_{P}. \end{array} \end{array}$$

If Δ*t* ≤ *T*_0_, the synapse undergoes depression through multiplicative LTD:

$$\begin{array}{c} G_{ij}\to G_{ij}-\{\begin{array}{cc} A_{D}G_{ij}\left(T_{0}-\Delta t\right)/T_{D}, & \text{if}\;\Delta t>T_{0}-T_{D},\\ A_{D}G_{ij}\exp\left(\left(\Delta t-T_{0}+T_{D}\right)/\tau_{D}\right), & \text{if}\;\Delta t\leq T_{0}-T_{D}, \end{array} \end{array}$$

The following parameters were used in simulations unless specified: *A*_*P*_ = 0.25 nS, *A*_*D*_ = 0.02, *T*_0_ = 2 ms, *T*_*P*_ = 3 ms, *T*_*D*_ = 3 ms, *τ*_*P*_ = 30 ms, *τ*_*D*_ = 30 ms. All weights were clipped below *G*_*min*_ = 0 nS and above *G*_*max*_ = 4 nS.

### Synapse states

Synapses were in 1 of 3 possible states depending on their synaptic weight. Synapses with weights 0 < *W* < *W*_*a*_ were silent and did not elicit response in postsynaptic neurons. Synapses with weights *W*_*a*_ < *W* < *W*_*s*_ were active and produced depolarization in postsynaptic neurons. Synapses with weights *W* > *W*_*s*_ were supersynapses that produced a strong response in postsynaptic neuron. Regardless of their state, all synapses participated in BTDP update rules. The following parameters were used in simulations unless specified: *W*_*a*_ = 0.2 nS, *W*_*s*_ = 1.0 nS.

### Potentiation decay

All synapses experience a depression at the end of each trial: *G* → *G* − *δ*, where *δ* = 0.01 nS. This depression is needed to prevent the emergence of too many active synapses that may lead to uncontrolled network growth [29].

### Axon remodeling

The axon remodeling rule was identical to the one in [28]. When the number of efferent supersynaptic connections of a neuron reaches *N*_*s*_ = 10, the neuron is saturated and all other active efferent connections of the neuron are withdrawn. Withdrawn connections do not elicit effect on postsynaptic neurons and do not participate in BTDP updates. However, they still undergo potentiation decay. Withdrawn connections will be re-connected if the neuron loses one or more of its supersynapses.

### Neural activity analysis

Burst density was calculated as a histogram of burst onset times with bin size 1 ms. The presence of oscillations in burst density was estimated using the coefficient of variation (CV), which is a standard deviation divided by the mean. Jitter in a neuron’s timing was calculated as a standard deviation of the burst onset times based on the 200 test runs of the dynamics of the grown network.

### Network structure

Plots of network topology were based on the supersynaptic weights between neurons and were created using Kamada-Kawai algorithm in Pajek software program for network analysis [47].

Network structure was also analyzed using the similarity of inputs to neurons that spike synchronously within a time window *T*_*w*_. For neuron *i* that bursts at *t*_*i*_, the synchronously spiking neurons have their burst onset times within a time interval (*t*_*i*_ − *T*_*w*_/2, *t*_*i*_ + *T*_*w*_/2). The similarity of inputs to neuron *i* and a synchronously spiking neuron is computed as the fraction of the presynaptic neurons common to the two neurons among all presynaptic neurons to the two neurons (the Jaccard index). The mean Jaccard index of all synchronously spiking neurons at *t*_*i*_ represents the similarity of inputs at this time. The mean Jaccard index for all burst times is defined as the similarity of inputs for a given time window *T*_*w*_.

### Analysis of inhibition

With the neuronal turnover disabled and the conduction velocity set to 100 *μ*m/ms, inhibitory conductance of all HVC_RA_ neurons was tracked for 30000 trials. By the end of these trials, the number of supersynaptic and active connections have reached stable values and the network growth stopped. A neuron was designated as recruited if it spiked consistently during the testing trials of the grown network in more than 95 out of 100 trials. The time of its recruitment was estimated using its spike history during the growth. At each trial, the number of the neuron’s spikes averaged over a window of the past 25 trials was computed, and when the average first reached 1, which signaled the start of reliable spiking, the trial was defined as the trial at which the neuron was recruited.

For a recruited neuron *i*, an LTP window is defined relative to the burst time of its presynaptic neuron *j*, during which the synaptic strength from neuron *j* to neuron *i* can be strengthened according to the BTDP rule. Specifically, the window is the time interval (*t*_*j*_ + *d*_*ji*_ + *T*_0_, *t*_*j*_ + *d*_*ji*_ + *T*_0_ + *τ*_*P*_), where *d*_*ji*_ is the axonal delay; *T*_0_ = 2 ms is the time shift in the BTDP rule; and *τ*_*P*_ = 30 ms is the time scale of the LTP part of the BTDP. At each trial before the recruitment, a set of inhibitory conductance traces on neuron *i* is extracted in the LTP windows relative to all its presynaptic neurons. The average of this set represents an inhibitory conductance of the recruited neuron at trial *T* aligned to its presynaptic neurons. For comparison, an average inhibitory conductance of the non-recruited neurons is extracted in the same time intervals, and is defined as the inhibitory conductance of the non-recruited neurons. Difference in the area under conductance curves is computed numerically using a trapezoid method. The median difference in the area computed for all trials before the recruitment represents the difference in the inhibitory conductance between the recruited neuron and the non-recruited neurons.

For analysis of inhibition on a recruited neuron *i* relative to its own burst onset times before the recruitment, only trials in which neuron *i* produced bursts are considered. For each such trial, the area under the inhibitory conductance curve is calculated for 10 ms before and 10 ms after the burst onset time. The median of the difference in area for all trials represents the difference in the inhibitory conductance before and after bursting of neuron *i*. The difference of the inhibitory conductance before burst relative to the average is defined as median of the differences between the mean inhibitory conductance 10 ms before the burst and the mean during the trial for all trials before the recruitment.

To investigate the inhibition after recruitment, similar procedure is applied to 100 test trials of the grown network.

## Supporting information

S1 Fig Neuronal age and spontaneous activity. In the model, spontaneous firing rate of HVC_RA_ neuron decreases with neuronal age due to reduced excitability.
(EPS)

S2 Fig Comparison of recruited (green lines) and non-recruited neurons (blue lines). For a recruited neuron, the number of bursts in each trial quickly stabilizes to 1 after the recruitment, and the total excitatory conductance to the recruited neuron jumps to a large stable value as well. In contrast, for a non-recruited neuron the number of bursts in each trial fluctuates at small values, and gradually decreases, until the neuron is replaced by a new immature neuron. The total excitatory conductance to the non-recruited neuron also fluctuates at small values and gradually decreases due to potentiation decay.
(EPS)

S3 Fig Comparison of inhibitory conductance for a grown network based on 100 test trials. A: Difference in the area under the conductance curve in the LTP window (Δ) for all recruited neurons aligned to presynaptic parents. This corresponds to Fig 8D and 8E in the main text. Δ is significantly negative (*p* < 10^−26^, Wilcoxon signed-rank test). B-C: Analysis of inhibitory conductance of recruited neurons aligned post-synaptically. B: Difference in inhibitory conductance after and before burst for all recruited neurons. This corresponds to Fig 8F and 8G in the main text. *A*_before_ and *A*_after_ are the sum of the inhibitory conductance within 10 ms window before and after the burst. *A*_before_ − *A*_after_ is much smaller than during the chain growth, but is still significantly positive (*p* < 10^−5^ Wilcoxon signed-rank test). C: Difference in inhibitory conductance 10 ms before burst and the mean inhibitory conductance during the trials for all recruited neurons. This corresponds to Fig 8J in the main text. The difference is significantly positive (*p* < 10^−50^ Wilcoxon signed-rank test) because interneurons fire with much higher rates during the network activity than during the entire trials on average.
(EPS)

S4 Fig Loop formation in the network with noisy mature HVC_RA_ neurons. When we use a single population of mature spontaneously active HVC_RA_ neurons receiving a large white noise stimulus of amplitude 0.25 nA to soma and 0.5 nA to dendrite, loop sequences form. Here we use a fast conduction velocity 1000 *μ*m/ms, which leads to the emergence of a synfire chain. A: Raster plot of network dynamics. B: Network topology based on synaptic weights between neurons.
(EPS)

S5 Fig Example HVC_RA_ neuron recorded in the subsong stage showing tight burst without being locked to the subsong onset. (Left) Firing rate of the neuron aligned to syllable onset times does not show significant peak, meaning that the neuron is not locked to the syllables. (Right) Example extra-cellular recording traces of the same neuron demonstrate a tight bursting pattern.
(EPS)
